# Water management for Power-to-X offshore platforms: an underestimated item

**DOI:** 10.1038/s41598-023-38933-w

**Published:** 2023-07-28

**Authors:** Yair Morales, Prantik Samanta, Fadi Tantish, Harald Horn, Florencia Saravia

**Affiliations:** 1 DVGW-Research Center at the Engler-Bunte-Institut, Water Chemistry and Water Technology, Karlsruhe Institute of Technology, Engler-Bunte-Ring 9, 76131 Karlsruhe, Germany; 2 Karlsruhe Institute of Technology, Engler-Bunte-Institut, Water Chemistry and Water Technology, Engler-Bunte-Ring 9, 76131 Karlsruhe, Germany

**Keywords:** Engineering, Environmental sciences, Energy science and technology

## Abstract

Increasing carbon dioxide (CO_2_) concentration in the atmosphere is considered one of the most important challenges today. Therefore, capturing CO_2_ and producing alternative energy sources through Power-to-X (PtX) approaches have become relevant scientific topics in recent years. However, there is a significant research gap regarding water management in PtX processes, particularly in offshore operations. The present study evaluates relevant aspects and possible challenges with respect to water management as well as mass and energy balances in conceptual offshore methane and methanol production platforms. The results show that 1600 m^3^ of seawater must be desalinated to supply the electrolyzer and reach a daily 50-Megagram (Mg) hydrogen production. Around 1100 m^3^ of brine coming out of the desalination plant may be discharged to the sea as long as prior environmental impact assessments are conducted. Additionally, 273 Mg and 364 Mg CO_2_ need to be generated daily by direct air capture to produce 99 Mg day^−1^ methane and 265 Mg day^−1^ methanol, respectively. The daily produced methane and methanol wastewater is estimated to be 223 and 149 m^3^, respectively. Based on the scant literature on methanol wastewater, this is expected to contain toxic substances. Zero liquid discharge (ZLD) is proposed as wastewater method. The corresponding energy demand for the water management facilities is projected to be negligible compared to the other PtX processes. The presented management of water streams in PtX platforms would not only help recover some of the resources (water, hydrogen and methanol), but also substantially contribute to the production cycle itself while leading toward a more sustainable approach.

## Introduction

The current emission of anthropogenic carbon dioxide (CO_2_) together with its increasing concentration in the atmosphere and oceans is certainly one of the most challenging global issues today^1^. A recent report from the Intergovernmental Panel on Climate Change shows that, among all major groups of greenhouse gases, CO_2_ holds the largest share of global net emissions from anthropogenic sources (around 75% in 2019)^2^. The Paris Agreement of the United Nations Framework Convention on Climate Change came to the light to establish a road map aiming to reduce CO_2_ emissions from fossil fuel burning and limit global warming to well below 2 °C^3^. As a result, several approaches as well as governmental incentives and strategies have been established to promote and develop renewable energies and concepts such as Power-to-X^4–7^. Power-to-X (PtX) consists in the production of hydrogen (Power-to-Hydrogen)^8^ or other clean fuels and energy carriers, such as methane, methanol and other synthetic fuels^9–12^, with the use of electricity coming from wind or solar energy, among other renewable sources. Offshore platforms are considered as potential areas of implementation for PtX technologies as they provide optimal conditions in terms of availability of existing infrastructure and resources such as water, CO_2_ and renewable energy^6,13^. In order to move away from fossil fuels and reduce the carbon footprint, an improvement in the efficiency of energy production is fundamental. One major issue of renewable sources, such as wind energy, is their fluctuating operation. However, the generation of energy carriers in the form of PtX products offers a way to easily store and transport surplus or intermittent electricity^14,15^.

Offshore Power-to-X platforms generally comprise of seawater desalination followed by a water electrolysis unit to produce pure hydrogen^8^. Hydrogen can be used as fuel or further processed along with CO_2_ in a synthetic fuel unit, where a final product and wastewater are generated. The most relevant units and technologies for PtX production of methane and methanol are presented hereafter.

Currently, hydrogen is largely produced from fossil fuels; a source that not only is unsustainable but generates significant CO_2_ emissions^16^. Recent advances in alternative technologies, such as water electrolysis, have allowed the use of renewable energies for emissions-free hydrogen generation. Electrolysis involves the splitting of water molecules into hydrogen and oxygen by applying an electrical current.

Alkaline electrolyzers (AEL), proton exchange membrane (PEM) electrolyzers, and solid oxide electrolyzers (SOEL) are some examples of water electrolysis technologies. In principle, water electrolysis consists of two electrodes submerged in or separated by an electrolyte^4^. AEL and PEM are advanced and commercially available technologies, as opposed to SOEL, which is still in early demonstration phases^16^. Comprehensive studies from the International Renewable Energy Agency^4,16^ provided a comparison between AEL and PEM along with forecasts on their performance and techno-economic characteristics. The studies presented the advantages that make PEM a more suitable technology for offshore operations over its commercial counterpart; including its fast response times, reduced overall footprint and flexible operation^4,16,17^. PEM electrolyzers are not built to operate directly with seawater but require ultra-pure water with a feed quality below 0.5 ppm total dissolved solids (approximately 1 μS cm^−1^)^17,18^. In consequence, any unreacted water which is internally recirculated must be regularly removed and retreated to avoid concentration of substances within the electrolyzer. In offshore environments, such high quality can be achieved by means of desalination technologies.

Reverse osmosis (RO) is currently the main commercially-available technology for the production of fresh water from brackish and seawater^19,20^. RO is a pressure-driven process in which water is pumped through a series of semi-permeable membranes, thereby generating a low salinity product (permeate) along with a residual concentrated brine (concentrate). Apart from the pressurized RO membrane modules, RO plants may include a pretreatment step consisting of filtration and addition of chemicals to protect the RO membranes, as well as a post-treatment to adjust and reach the target quality. The required water quality for PEM electrolysis cannot be achieved with RO alone, thus a polishing post-treatment, typically through ion exchange or electrodialysis, is needed. RO entails electricity to achieve high operating pressures. As a way to fulfill the energy demand for these systems and take advantage of renewable sources, membrane desalination plants have been coupled with renewable energies in the recent years^21^. For instance, Serna and Tadeo^22,23^ simulated the operation and integration of RO desalination with both fluctuating wave energy and PEM electrolysis. Other desalination technologies, such as electrodialysis, have also been shown to be suitable alternatives for fresh water supply with variable power sources (hybrid photovoltaic/wind)^24^.

Methane is an energy carrier of significant importance to the industry, energy, and transportation sectors worldwide. Two major approaches exist to produce synthetic methane: biological methanation and catalytic methanation. Catalytic methanation involves the hydrogenation of either carbon monoxide (CO) or CO_2_ to produce methane and water^14,25^. Catalytic reactors, in particular fixed-bed reactors, are considered the state of the art for large-scale applications^26^. In the Power-to-X concept, CO_2_ from point sources or air is the target feedstock along with hydrogen produced by electrolysis. From a stochiometric standpoint, CO_2_ methanation generates twice as much wastewater—which must be handled before disposal or reuse—for every mol of methane produced compared to CO methanation. In addition, both approaches are largely exothermic and therefore heat management is required.

Methanol is used as an energy carrier for hydrogen storage and transportation and has several applications in the chemical industry^27–29^. Currently, 90% of the total methanol is produced from natural gas through high-pressure (the BASF process) and low-temperature methods^30^. Additional available approaches include methanol production from coal, biomass^31^ and the catalytic hydrogenation of CO_2_^32^. Similar to methane synthesis, the generation of methanol through green hydrogen and CO_2_ is considered as a suitable and promising approach for PtX. Likewise, the reaction of hydrogenation of CO_2_ to methanol is slightly exothermic and requires cooling as well as generates wastewater as by-product.

In offshore environments, the generation of methane and methanol wastewater together with a large brine production after desalination through RO have the potential to be an environmental bottleneck of these operations. Research has shown that slight changes in salinity and temperature caused by brine disposal practices may have an adverse impact on marine ecosystems^33^. A previous study on the feasibility of offshore desalination highlighted the environmental impact as an important aspect to consider in platform-based facilities^34^. Water intake, backwash/concentrate neutralization and strategic discharge are of high relevance. To the best of the authors’ knowledge, there are no studies in the current literature that provide information on the characteristics or the precise management of process wastewaters from catalytic methanation reactors. A single study by Schirrmeister et al.^35^ has reported that wastewater from a methanation demonstration plan was processed and reused for water electrolysis without further details. Likewise, literature about methanol wastewater is highly limited. Methanol wastewaters are considered as toxic and proven to negatively impact the human and ecological environment^36,37^. Table 1 summarizes the characteristics of wastewater coming from methanol synthesis. The wide range of values shows the variability of the reported qualities, depending on the production process. Apart from the substances listed in Table 1, formaldehyde and methanoic acid have also been observed in methanol wastewater^36^.

**Table 1:** Characteristics of low concentration methanol wastewater^36,37^.

Parameter/substance	Values	Units
Chemical oxygen demand	1–40	g L^−1^
Suspended solids	281	mg L^−1^
Ammonium nitrogen	0.33	mg L^−1^
Total phosphorus	0.03	mg L^−1^
pH	4.8–7.7	–
Methanol	350–2000	mg L^−1^

Therefore, a sustainable wastewater treatment is the need of the hour. Zero liquid discharge (ZLD) has previously been considered for treating brine^17,38^ as well as wastewater^39^. Conventional ZLD approaches consist of a series of thermal processes, often in combination with membrane technologies^40^. Although ZLD is becoming very popular in industrial wastewater treatment in several countries, the energy consumption and the implementation cost remain very high. However, globally increasing freshwater scarcity and the rising cost of wastewater disposal made ZLD an attractive water treatment and management strategy^41^. Hence, ZLD approach was followed in this research study.

Currently, there is a lack of literature that incorporates water management as a main topic of Power-to-X. Available publications on inland or offshore water electrolysis^42,43^ and/or generation of methane, methanol and other PtX products^12,13,44^ primarily limit their focus on the processes themselves, their integration or modelling. Hence, the management, disposal or treatment of water are barely addressed^17^ or not mentioned. Nevertheless, water is highly relevant in PtX operations as it is an important input and the main byproduct. Particularly in remote locations like offshore platforms, a proper and self-sufficient water management is essential. This work looks into the relevant aspects that surround the use and handling of water on offshore PtX platforms and how these compare to other processes in terms of energy consumption. Additionally, this study aims to provide an initial overview of the mass balance and different water streams in such operations by evaluating a conceptual PtX platform with a daily 50-Megagram (Mg) hydrogen production in which methane and methanol are considered as two separate target product scenarios.

## Methods

### Offshore platform concept

**Figure 1:**
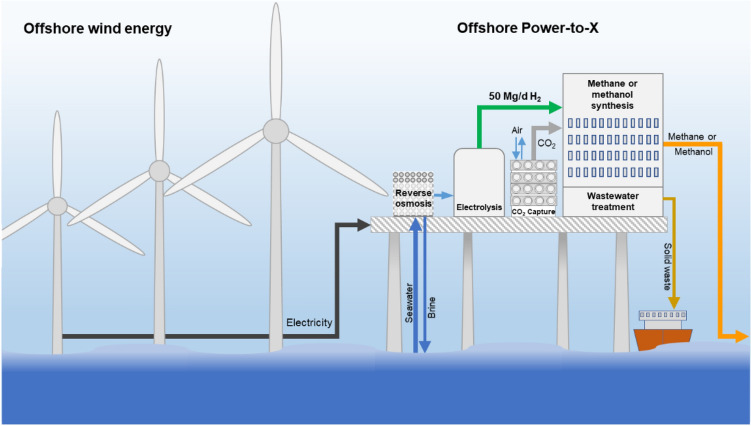
Illustration of the conceptual wind-powered PtX platform for methane or methanol production.

The selected location for the conceptual offshore Power-to-X platform is the German Bay region in the North Sea. Two production scenarios were selected for the study: one platform for methane and another one for methanol as final product. The results presented in this work were performed with the assumption that energy is supplied by offshore wind parks without interruption or varying loads, so the entire platform was in continuous operation. A production of 50 Mg of hydrogen a day from the electrolyzer was defined as the base size of the platform. Accordingly, the design and estimation of all processes in the two scenarios were derived from this parameter. Figure 1 shows a graphical depiction of a wind-powered PtX platform, in which seawater is pumped into a desalination step to later be used to produce hydrogen and methane or methanol from CO_2_ in catalytic reactors. In this concept, wastewater from the synthesis process is treated to avoid its disposal back into the sea. The only outputs of the platform are brine from the desalination, solid waste and the target product.

### Seawater desalination

A desalination plant was designed to determine the input water quantity needed to supply the electrolyzer with ultra-pure water as well as to estimate the energy demand of the overall desalination process. The plant consists of pretreatment, desalination through reverse osmosis and post-treatment with ion exchange. The desalination process was simulated in Water Application Value Engine (WAVE, DuPont) to estimate the recovery rate and specific energy consumption. Feed seawater composition and conditions for the design were taken from available literature from the selected offshore location. The permeate required to cover the desired daily hydrogen production was estimated by the general chemical reaction in an electrolyzer:

1 $$2{H}_{2}O\left(l\right)\to {2H}_{2}\left(g\right)+{O}_{2}\left(g\right) \;\; \Delta H\text{ = +285.8 } \mathrm{kJ}\; {\mathrm{mol}}^{-1}$$

with the assumption that 90% of the water is split, as described for commercial electrolyzers^45^, while the unreacted 10% is returned to the ion exchange system. Additional water demands for cooling or steam generation in the other processes was not considered for the design since data on the required quantities and qualities are limited in literature. Moreover, cooling systems in the different processes may also be operated with locally abundant seawater or cooling oil and not strictly with purified water.

### Electrolysis

The rated power ${P}_{el,PEM}$ [W] for the electrolyzer was calculated by Faraday’s law^46^:

2 $${P}_{el,PEM}=\frac{{\dot{n}}_{{H}_{2}}\cdot z\cdot F\cdot {U}_{el}}{{\eta }_{F}},$$

where ${\dot{n}}_{{H}_{2}}$ is the amount of hydrogen produced [mol s^−1^], $z$ is the valence number of ions of hydrogen (2), $F$ is the Faraday constant and ${\eta }_{F}$ the faraday efficiency (up to 99%^46^). The applied cell voltage ${U}_{el}$ has to exceed the thermoneutral voltage needed to split a water molecule (1.48 V)^18^. Commercial electrolyzers are typically operated between 1.6 to 2 V depending on the current density and operating temperature; a low current density is equivalent to a low voltage but results in lower production rates and a larger electrolyzer^47^. Since space is a limiting aspect in offshore platforms, a voltage of 2 V was used in this study. The assumed operating pressure and temperature were 30 bar and 80 °C.

### Direct air capture (DAC)

DAC using solid-based adsorption and desorption was considered in this study because it requires lower temperature, approximately 80–100 °C which allows the integration of waste heat from electrolyzers and synthetic fuel synthesis. Commercial solid-base DAC can capture 0.14 Mg CO_2_ day^−1^ while consuming 2.5 MWh_th_ thermal energy per Mg CO_2_ and 0.5 MWh_el_ per Mg CO_2_^48^.

The theoretical amount of CO_2_ [g] needed for either methane synthesis or methanol synthesis can be determine based on Eq. (3). The ideal stoichiometric ratios ($SR$) of H_2_/CO_2_ are 4 and 3 for methane and methanol, respectively. The governing parameter here is the daily amount of H_2_ produced by the electrolyzer, ${n}_{{H}_{2}}$ [mol].

3 $${m}_{{CO}_{2}}=\frac{{n}_{{H}_{2}}}{SR}\times {MW}_{{CO}_{2}},$$

where ${MW}_{{CO}_{2}}$ is the molecular weight of CO_2_. The resulting mass of carbon dioxide ${m}_{{CO}_{2}}$ was then used to estimate the necessary electrical and thermal energy demand.

### Methane synthesis

Fixed-bed methane synthesis based on honeycomb catalysts was selected for this study due to its good load flexibility^26^. The methanation process was adapted from a demonstration plant operated by Mörs et al.^49^ to convert H_2_ and CO_2_ to methane (CH_4_) as described by the Sabatier reaction:

4 $${CO}_{2}+{4H}_{2}\leftrightharpoons {CH}_{4}+{2H}_{2}O \;\; \Delta H =-165 \; \mathrm{kJ }\; {\mathrm{mol}}^{-1}.$$

The two-phase methanation consisted in a reactor with multi-tube channels coated with a catalyst followed by a polishing reactor. The total energy demand of the methane synthesis was taken as 1.2 MWh_el_ per Mg CH_4_. Assuming 100% conversion of hydrogen, the daily estimated methane production [g] was calculated using Eq. (5).

5 $${m}_{{CH}_{4}}=\frac{{n}_{{H}_{2}}}{SR}\times {MW}_{{CH}_{4}},$$

where $SR$ equals 4, and ${MW}_{{CH}_{4}}$ is the molecular weight of methane.

### Methanol synthesis

Methanol synthesis through catalytic hydrogenation of CO_2_ methods were considered in this study and estimated by following the proposed offshore model from Patterson et al.^12^, where Eq. (6) was considered as the main production formula^50^.

6 $${CO}_{2}+{3H}_{2}\leftrightharpoons {CH}_{3}OH+{H}_{2}O \;\; \Delta H =-49.4 \; \mathrm{kJ}\; {\mathrm{ mol}}^{-1}$$

The stoichiometric ratio ($SR$) of H_2_/CO_2_ was calculated as 3. Therefore, ideally 1 mol of CO_2_ and 3 mol of hydrogen are needed to produce 1 mol of methanol (CH_3_OH) and 1 mol of water. With 100% conversion rate of the reactants, the daily estimated methanol production [g] was calculated by Eq. (7).

7 $${m}_{{CH}_{3}OH}=\frac{{n}_{{H}_{2}}}{SR}\times {MW}_{{CH}_{3}OH},$$

where ${MW}_{{CH}_{3}OH}$ is the molecular weight of methanol.

### Wastewater treatment

Considering the conversion assumptions followed for methane and methanol production, the daily volume of wastewater [m^3^] from each synthesis processes was estimated as follows:

8 $${V}_{WW, PtX}=\frac{{n}_{{H}_{2}}}{SR\times {10}^{6}}\times {MW}_{{H}_{2}O }$$

in which ${MW}_{{H}_{2}O}$ is the molecular weight of water, and $SR$ was taken as 2 and 3 for methane and methanol wastewater, respectively.

ZLD was considered for the calculations as the worst-case scenario in terms of energy consumption. The calculation approach for energy demand of the ZLD process was focused on treatment of the methane and methanol wastewaters, followed by reuse of the recovered water. A normalized energy demand of 58.6 kWh_el_ m^-3^ was taken into consideration for methane and methanol wastewater treatment as assumed in literature for wastewater treatment through conventional thermal ZLD technologies^39^. The energy consumption by ZLD of the respective wastewater volumes was calculated as follows:

9 $${E}_{ ZLD, PtX}={V}_{WW, PtX}\times 58.6.$$

## Results

**Figure 2:**
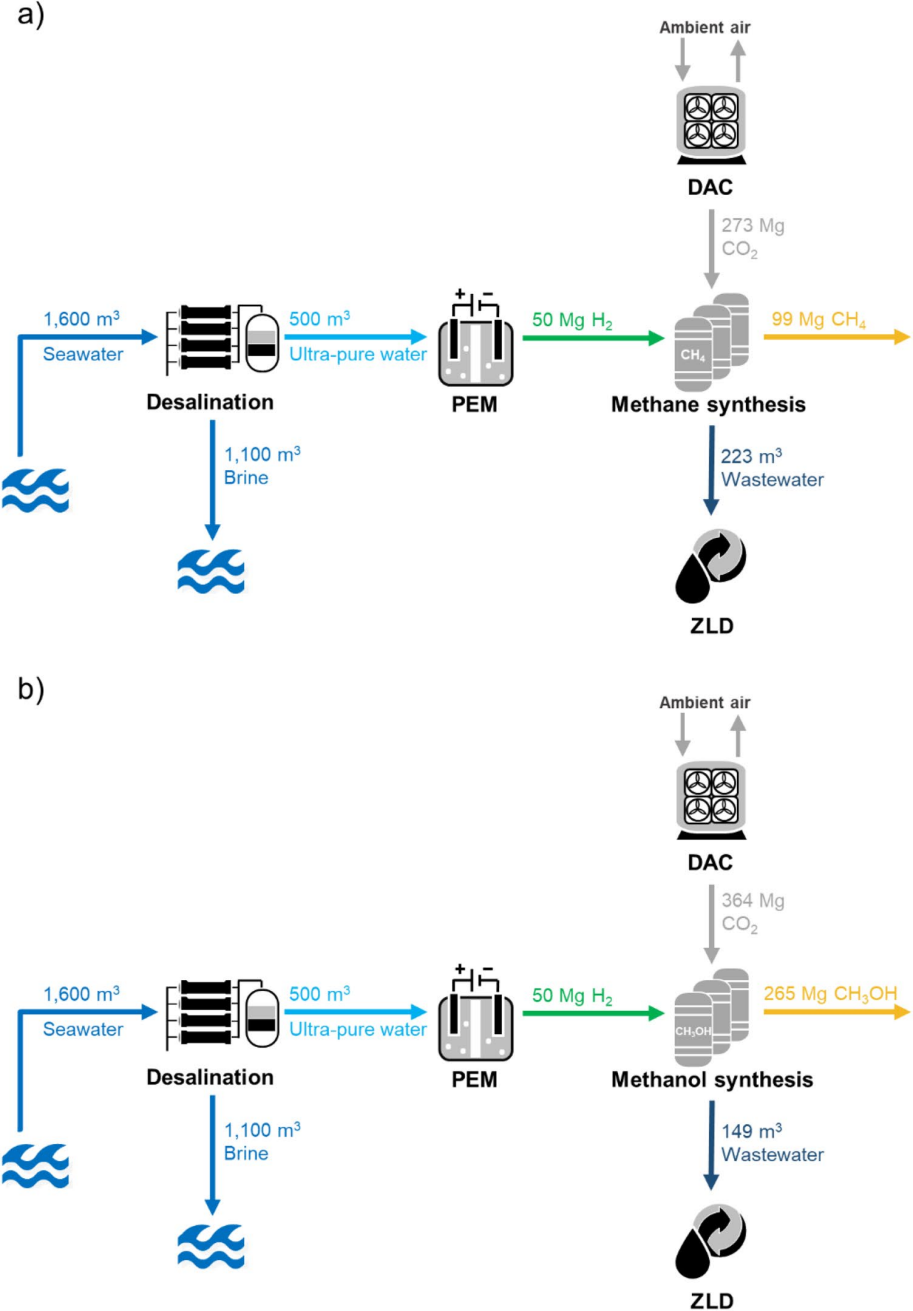
Schematic of PtX platforms for production of (**a**) methane and (**b**) methanol.

The process chains of the PtX platform are depicted in Fig. 2. Likewise, the figure summarizes the main daily inputs and outputs of each process unit for both production scenarios (i.e., methane and methanol as final products).

### Desalination

As seen in Fig. 2, the desalination plant treats under average conditions around 1600 m^3^ of seawater per day at an overall recovery of around 32%, thus delivering 500 m^3^ day^−1^ of ultra-pure water (< 0.1 μS cm^−1^) to the electrolyzer. The plant was designed to operate in a two-pass mode in order to reduce the amount of chemicals required for the post-treatment and maintain offshore storage and disposal challenges to the minimum. The energy consumption of the reverse osmosis system was estimated at 5.9 kWh_el_ per m^3^ of produced water, while the consumption for the ultrafiltration pretreatment and post-treatment through ion exchange mixed beds was 0.2 and 0.1 kWh_el_ m^−3^, respectively, resulting in a total specific energy consumption of is 6.2 kWh_el_ m^−3^.

Around 1100 m^3^ of brine are produced daily by the desalination plant and returned to the sea. This stream contains a salinity level higher than the local conditions of the North Sea along with additional treatment chemicals. Concentrate discharge into the open-ocean is a common disposal practice in coastal desalination plants and may be a solution in offshore operations^19^. The Oslo-Paris Convention for the protection of the marine environment of the North-East Atlantic (OSPAR) strictly monitors and regulates the disposal of waste streams in offshore oil and gas installations. Thus, OSPAR regulations may apply for chemicals contained in the brine depending on their toxicity and biodegradability, however, the Convention currently does not state any discharge limits for salinity levels^51^. Nevertheless, an effective disposal management would certainly require site-specific assessments and the proper discharge equipment (i.e., piping and diffuser systems) to reduce its environmental impact as much as possible^52,53^.

### Electrolysis

The PEM electrolysis system was designed to operate at 111.9 MW_el_ in order to produce 50 Mg of hydrogen per day. This estimates that around 54 kWh_el_ are needed to produce 1 kg of hydrogen, which corresponds to current energy demand values reported for PEM plants of similar magnitudes^17,42,54^. Moreover, the efficiency of the plant lies within the typical range and was found to be 62% with respect to the lower heating value of hydrogen^16,17,54^.

### DAC

A daily production of 50 Mg of hydrogen corresponds to 24.8 × 10^6^ mol of hydrogen. The required amount of CO_2_ to be capture and provided for synthetic fuels synthesis along with the energy demand required to capture this amount are shown in Table 2.

**Table 2:** Carbon dioxide feed mass rate and required thermal and corresponding electrical energies for capturing.

	Methane synthesis	Methanol synthesis
Required feed CO_2_ (Mg CO_2_ day^−1^)	273	364
Total thermal energy demand (MWh_th_ day^−1^)	682.5	910
Total electricity demand (MWh_el_ day^−1^)	136.5	182

The total estimated energy demand in this study is 3.0 MWh Mg^−1^ CO_2_, which is slightly higher than the reported DAC industrial plant energy requirements from 1.8 to 2.45 MWh Mg^−1^ CO_2_^55^.

### Methane synthesis

Based on the ideal hydrogenation of CO_2_ in Eq. (4), the conversion of 273 Mg CO_2_ day^−1^ with 50 Mg H_2_ day^−1^ produces a maximum of 99 Mg CH_4_ day^−1^ and 223 m^3^ day^−1^ CH_4_ wastewater (Fig. 2a) as given by Eqs. (5) and (8), respectively. The total energy demand was estimated at 120 MWh_el_ day^−1^.

### Methanol synthesis

The total conversion of 364 Mg CO_2_ day^−1^ with 50 Mg H_2_ day^−1^ produces 265 Mg CH_3_OH day^−1^ and 149 m^3^ day^−1^ CH_3_OH wastewater (Fig. 2b) by Eqs. (7) and (8), respectively. The electrical and thermal energy inputs in addition to the thermal energy output during methanol synthesis were adapted from the details shown in Fig. S6 of the supplementary information from Patterson et al.^12^. The calculated required electrical power consumed by the compressors was found 0.74 MW_el_ while the thermal energy input was mainly required by the reboiler of the distillation column which requires 1.0 MW_th_ per 1 Mg methanol production. These consumptions corresponded to approximately 196 MWh_el_ and 265 MWh_th_. The thermal energy output equaled to the sum of thermal energies produced by compressors, steam drum, heat exchangers, hydrogen purge, and distillation column which resulted in 3.1 MW_th_. 1.0 MW_th_ of the produced thermal energy was reused for the reboiler.

### Wastewater treatment

**Figure 3:**
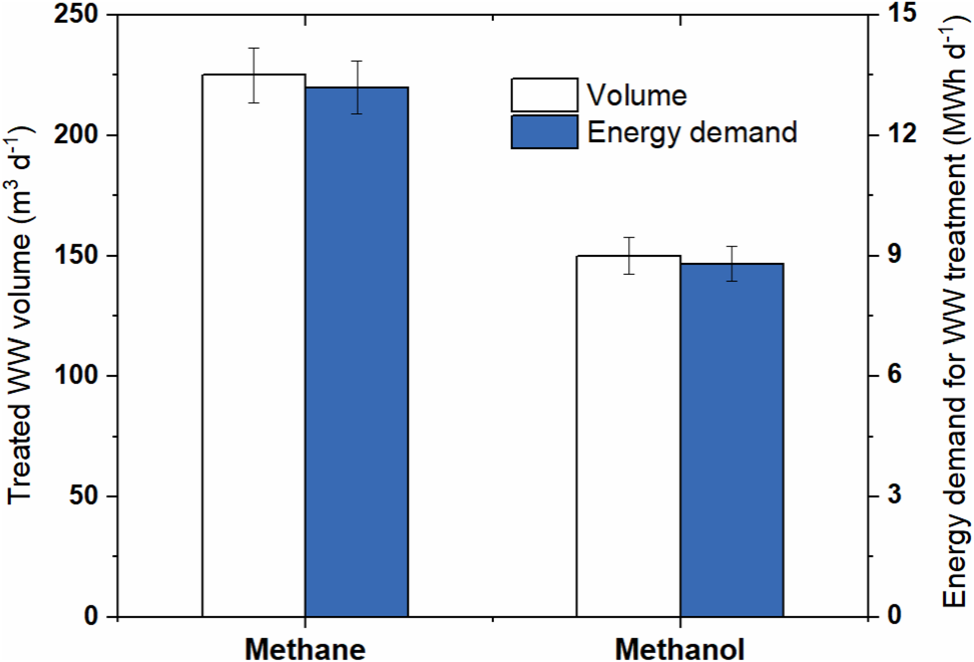
Volume of wastewater from methane and methanol production per day and corresponding energy demand for treatment with zero liquid discharge.

The methane and methanol wastewater volume per day was calculated by following Eq. (8). Consequently, the daily required energy to treat methane and methanol wastewater was calculated by Eq. (9). The daily methane wastewater production was found 33.3% higher than the daily produced methanol wastewater. This led to consume 1.5 times higher energy to treat the methane wastewater as well (Fig. 3).

## Discussion

As seen in Fig. 2, coupling methanol synthesis with a base daily production of 50 Mg of hydrogen would result in a larger amount of PtX product than having methane as final product. However, the total energy content of the expected production of methanol is just slightly higher than for methane despite the lower product quantity—namely, 1376 MWh for 99 Mg day^−1^ CH_4_ and 1479 MWh for 265 Mg day^−1^ CH_3_OH. The final products would need to be transported to the coast.

**Figure 4:**
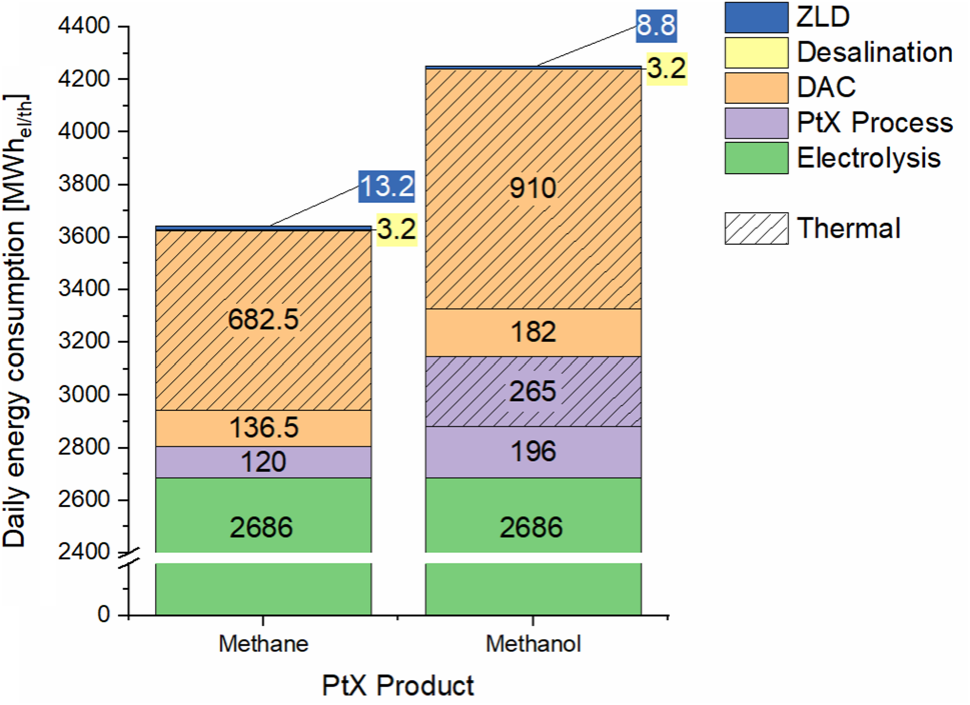
Daily energy demand of offshore platforms scenarios (methane and methanol production).

Moreover, a comparison of the cumulative daily energy consumption for both methane and methanol production platforms in Fig. 4 shows that the latter requires more energy to operate, as a consequence of its higher CO_2_ demand. In terms of electrical energy efficiency, there is no clear advantage between the two production scenarios. Around 47% of the input electrical energy in both process chains leaves in the form of the respective PtX product.

Electrolysis is the most energy demanding process of those evaluated, consuming nearly 87–91% of the total electrical energy. Whereas the desalination energy demands are practically negligible when compared to the rest of the processes with only 0.1%. This relatively low energy consumption has also been identified in Serna and Tadeo^23^ and Khan et al.^17^ showing the potential scalability of such a desalination treatment if additional water quantities are to be covered for heat management in other processes. Desalination is not expected to be significant in terms of energy demand regardless of the supply magnitudes of pure water required, but it may rather be limited by the space available as the plant itself and the required water and chemical storage capacity would be larger.

The treatment of the wastewater streams by ZLD required 0.3–0.5% of the total electrical energy (Fig. 4). From an energy perspective, the water management components in both production scenarios seem to be insignificant. Under variable electricity conditions, it may be expected that the operation will be adapted to the requirements of the other PtX processes.

**Figure 5:**
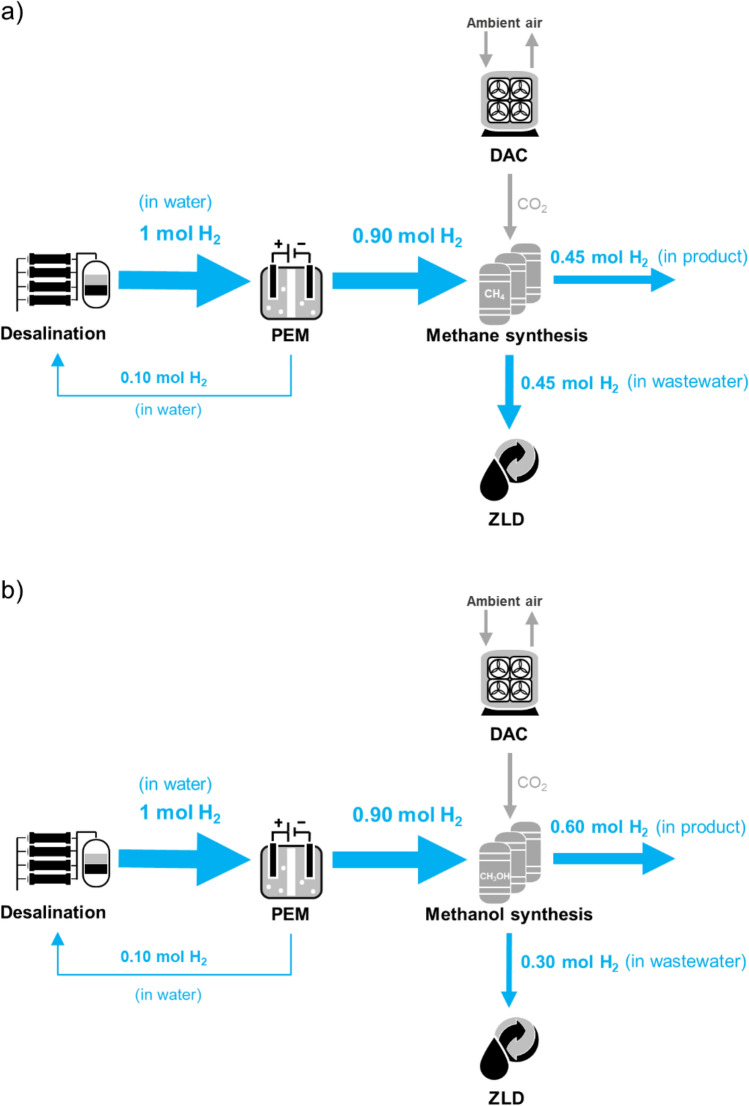
Estimated normalized mass flow for 1 mol of water as PEM input in the (**a**) methane and (**b**) methanol production platforms. Size of arrows represents the number of moles.

ZLD would be an important measure to recover a substantial amount of hydrogen in the form of wastewater. As depicted in the normalized flow diagram of hydrogen in the process chain (Fig. 5a,b), 0.45 mol and 0.30 mol of H_2_ per mol of water produced by the desalination plant are lost as wastewater from the methane and methanol production, respectively. This constitutes 33–50% of the initial hydrogen input into the PEM. Treating methanol wastewater by ZLD would also remove the substantial amount of COD and aromatics along with formaldehydes and methanoic acids that are considered as highly harmful to the ecological system and human health^37^. This measure would be in accordance with the OSPAR Convention regulations^51^. In addition, ZLD could allow for the recovery of residual methanol. For instance, it may be estimated that up to 61.25 kg CH_3_OH day^−1^ may be recovered when considering a methanol concentration of 350 mg L^−1^ in the wastewater stream^36,37^.

RO brine treatment would also provide further recirculation of hydrogen, considering around 70% of the feed water is returned to the sea. ZLD would be a potential strategy for this, however, its implementation on brine treatment and management is still expected to see a progressive growth^17,40^. Previous studies on brine management by ZLD have reported energy consumptions of conventional brine concentrators of up to 39 kWh m^−3^^41^, whereas conventional crystallizers may reach up to 70 kWh m^−3^^38^. Furthermore, the energy required for a complete treatment of the daily 1100 m^3^ brine by either of these technologies would still correspond to less than 3% of the consumption from the PEM electrolyzer. The application of ZLD as brine treatment method would bring back substantial amounts of pure water into the hydrogen production cycle as well as recover other valuable resources. This could have a massive potential to help the overall economy as well^17^. Nevertheless, in offshore environments, space and load capacities of the platform would be the final limiting factors.

The proposed method reflected that the most important greenhouse gas (CO_2_) can be captured from natural or industrial source, human activity, or air by absorption, and chemically transformed into methane and methanol^29^. In addition, the production of methane and methanol from CO_2_ can be regarded as a completely carbon–neutral process, considering that the hydrogen necessary for this productive cycle can be originated from water dissociation by electrolysis^56,57^ and renewable electricity is employed.

The presented results clearly indicate the staggering energy consumption related to overall brine and wastewater treatment. Nevertheless, it is quite negligible when compared with the energy demand related to PtX products generation itself. In addition, the potential recovery of methanol and reincorporation of product water from the synthesis processes would contribute to the circular approach of the economy. Although considering the energy demand of ZLD in this approach may present the maximum energy demand for the wastewater treatment, its substantial impact may not be ignored when moving towards more sustainable treatment approaches.

## Conclusion

A conceptual PtX platform for the generation of two separate product scenarios (methane and methanol) was evaluated to discuss the most important aspects that surround water management in offshore operations. Based on the results, a daily hydrogen production of 50 Mg from PEM electrolysis, together with 273 Mg and 364 Mg of captured CO_2_, can generate up to 99 Mg and 265 Mg of methane and methanol, respectively. It was estimated that the production chains would consume between 2945 and 3067 MWh_el_ (plus 683 and 1175 MWh_th_) on a daily basis and result in comparable efficiencies with respect to the energy content of the final products. In addition, the assessment of the presented PtX platform led to the following relevant remarks with respect to water management:

- The desalination step treats large amounts of water and around 70% comes out as brine. The brine contains the same local salt components as the raw water but at higher concentrations as well as treatment substances such as anti-scalants, cleaning agents and regeneration chemicals.
- Brine discharge was proposed as disposal approach and may be implemented in real operations. Such measures should still include the provisions that local regulations are followed and proper environmental assessments and disposal strategies are performed to evaluate and reduce the impact on marine environments.
- Between 30 to 45% of input hydrogen (in the form of water) into the PEM gets lost as wastewater during the methane and methanol generation processes. This is a reaction by-product from the hydrogenation of CO_2_ in both syntheses. However, data corresponding to wastewater volumes from real plants are not addressed in the available literature.
- While no information is available on the composition of methane wastewaters, only a few studies provided data on wastewater from methanol production. Methanol wastewater is of environmental concern and therefore shall not be directly discharged.
- Zero liquid discharge was proposed as the state-of-the-art solution to treat the waste streams in PtX-platforms as it provides the potential to eliminate all contaminants and ultimately reintegrate hydrogen as purified water into the processes.
- Although zero liquid discharge as well as desalination are associated with high energy demands, their respective estimated energy consumptions was practically negligible compared to the rest of the processes considered for the PtX platform. Space and weight still remain as possible limiting factors of the water management facilities in offshore operations.

Decarbonization is certainly an important goal today and a shift to renewable sources and PtX products is one clear way toward it. However, it is essential that aspects such as resources management (water) and environmental protection by waste control in offshore operations are sensibly considered in order to claim and ensure that these processes are as sustainable and green as possible.

## Data Availability

All relevant data analyzed during the current study are included in the manuscript. Raw data are available from the corresponding author upon reasonable request.
